# HMB/Arg/Gln may improve short-term outcomes after esophagectomy in patients with thoracic esophageal cancer

**DOI:** 10.1093/dote/doae121

**Published:** 2025-01-09

**Authors:** Katsushi Takebayashi, Sachiko Kaida, Reiko Otake, Asuka Fukuo, Toru Miyake, Masatsugu Kojima, Soichiro Tani, Hiromitsu Maehira, Haruki Mori, Hajime Ishikawa, Masaji Tani

**Affiliations:** Department of Surgery, Shiga University of Medical Science, Seta Tsukinowa-cho, Otsu, Shiga 520-2192, Japan; Division of Clinical Nutrition, Shiga University of Medical Science Hospital, Seta Tsukinowa-cho, Otsu, Shiga 520-2192, Japan; Department of Surgery, Shiga University of Medical Science, Seta Tsukinowa-cho, Otsu, Shiga 520-2192, Japan; Department of Surgery, Shiga University of Medical Science, Seta Tsukinowa-cho, Otsu, Shiga 520-2192, Japan; Department of Surgery, Shiga University of Medical Science, Seta Tsukinowa-cho, Otsu, Shiga 520-2192, Japan; Department of Surgery, Shiga University of Medical Science, Seta Tsukinowa-cho, Otsu, Shiga 520-2192, Japan; Department of Surgery, Shiga University of Medical Science, Seta Tsukinowa-cho, Otsu, Shiga 520-2192, Japan; Department of Surgery, Shiga University of Medical Science, Seta Tsukinowa-cho, Otsu, Shiga 520-2192, Japan; Department of Surgery, Shiga University of Medical Science, Seta Tsukinowa-cho, Otsu, Shiga 520-2192, Japan; Department of Surgery, Shiga University of Medical Science, Seta Tsukinowa-cho, Otsu, Shiga 520-2192, Japan; Department of Surgery, Shiga University of Medical Science, Seta Tsukinowa-cho, Otsu, Shiga 520-2192, Japan; Department of Surgery, Shiga University of Medical Science, Seta Tsukinowa-cho, Otsu, Shiga 520-2192, Japan

**Keywords:** Abound, Anastomotic leakage, Esophageal cancer, Esophagectomy, Hmb/arg/gln

## Abstract

**Background:**

The wound healing effects of a specialized amino acid supplement containing calcium beta-hydroxy-beta-methylbutyrate, L-arginine, and L-glutamine (HMB/Arg/Gln) have been reported. This study aimed to investigate the effectiveness of HMB/Arg/Gln in the perioperative management of patients with thoracic esophageal cancer.

**Methods:**

This retrospective cohort study included 131 patients who underwent esophagectomy for thoracic esophageal cancer between January 2016 and November 2023. Postoperative infectious complications (PICs) were compared between patients who received HMB/Arg/Gln for 7 days before surgery (n = 95) and those who did not (control group, n = 36).

**Results:**

Among the 111 male and 20 female patients (median age 68 years, range 38–84 years), stage I disease was found in 37 patients, stage II in 26, stage III in 61, and stage IVa in 7. Of the 131 patients, 36 (27.5%) had PICs, with PICs occurring in 20 (21%) of the HMB/Arg/Gln group and 16 (44.4%) of the control group. The PIC rate was significantly lower in the HMB/Arg/Gln than in the control group (p = 0.007). Propensity score matching analysis showed lower rates of anastomotic leakage (5.5% vs. 22.2%; p = 0.04) and Clavien–Dindo grade III or higher PICs (5.5% vs. 27.8%; p = 0.011) in the HMB/Arg/Gln than in the control group. The healing time for anastomotic leakage was shorter in the HMB/Arg/Gln (18 days, range 7–25 days) than in the control group (25 days, range 21–56 days) (p = 0.033).

**Conclusions:**

HMB/Arg/Gln supplementation was associated with reduced risk of anastomotic leakage and PIC severity following esophagectomy.

## INTRODUCTION

Esophageal cancer is generally regarded as an aggressive disease with a poor prognosis. Among patients with this cancer, the frequency of clinical stage I (cStage I) is approximately 20%, whereas clinical stage II/III (cStage II/III) (excluding cT4) accounts for approximately 50% of cases. All of these patients are candidates for esophagectomy, which remains the most important treatment option for esophageal cancer.^1^ Recent improvements in long-term survival after esophagectomy can be attributed to advancements in surgical techniques for extended lymph node dissection and perioperative management.^2^^,^^3^ However, esophagectomy is a highly invasive procedure associated with several serious postoperative complications, including pneumonia, recurrent laryngeal nerve palsy, and anastomotic leakage, which may result in multiorgan failure, death, and long-term morbidity among survivors.^4–7^

Several prophylactic strategies for postoperative complications have been implemented in esophageal cancer surgery. Nutritional management is important for patients with esophageal cancer. Effective nutritional support is necessary for those undergoing esophagectomy. An oral nutritional supplement composed of beta-hydroxy-beta-methylbutyrate, arginine, and glutamine (HMB/Arg/Gln) has been shown to improve wound healing by stimulating protein and collagen synthesis.^8^^,^^9^ This study aimed to evaluate whether HMB/Arg/Gln (Abound®) can prevent postoperative complications in patients who have undergone esophagectomy for esophageal cancer.

## METHODS

### Patients

We conducted a single-center retrospective cohort study of all patients who underwent radical esophagectomy. A total of 133 consecutive patients who underwent radical esophagectomy via right thoracotomy or thoracoscopic surgery (with cervical anastomosis) between April 2016 and November 2023 at Shiga University of Medical Science Hospital were included in this study. Patients with esophageal cancer who underwent planned esophagectomy and reconstruction with cervical anastomosis were included in this study. Two patients with R2 resection were excluded because of complications induced by remnant tumors. The remaining 131 patients were retrospectively analyzed. The tumor stage and pathological classification were assigned according to the Japanese classification of esophageal cancer.^10^ Nutritional status was evaluated using the Onodera prognostic nutritional index and the controlling nutritional status score, as previously described.^11^^,^^12^

### Nutritional support of HMB/Arg/Gln

Patients received a supplement of 48 g of HMB/Arg/Gln (2.4 g of HMB, 14 g of Arg, and 14 g of Gln) (Abound®; Abbott, Tokyo, Japan) dissolved in 200–400 mL of water for 7 days before surgery. This supplementation protocol has been consecutively implemented since October 2018. All patients received full HMB/Arg/Gln supplementation. In the control group, patients did not receive the HMB/Arg/Gln supplement preoperatively, either by their discretion (n = 8) or before the initiation of the protocol (n = 28).

### Surgical procedures

All surgeries were performed under general anesthesia with selective intubation to block the right lung. The procedures included a right transthoracic subtotal esophagectomy and lymph node dissection. Thoracic procedures were performed either through a right thoracic incision or via video-assisted thoracic surgery in the prone position. Abdominal procedures were conducted through an upper midline abdominal incision or by hand-assisted laparoscopic surgery. Cervical anastomoses were completed using either a circular stapler or hand sewing. Postoperatively, each patient was admitted to the intensive care unit. If the patient’s cardiopulmonary condition was stable, the patient was transferred to the general surgical ward on postoperative day (POD) 1. On POD 5, after evaluating the anastomosis using contrast medium by fluoroscopy, oral intake of thick liquids was initiated. This was gradually advanced to jelly-like food and then to solid food. Between April 2016 and November 2023, no aspects of perioperative management were changed, including the surgical procedure, postoperative use of antibiotics, perioperative nutritional support, and prehabilitation program.

### Postoperative complications

Postoperative complications were categorized using the Clavien–Dindo (CD) classification.^13^^,^^14^ In this study, pneumonia, wound infection, anastomotic leakage beyond CD classification grade II, and recurrent nerve palsy beyond CD classification grade I were considered postoperative complications. Anastomotic leakage, wound infection, and pneumonia were categorized as postoperative infectious complications (PIC). Patients were followed up from the date of esophagectomy until first outpatient visit.

### Statistical analysis

All statistical analyses were performed using EZR (Saitama Medical Center, Jichi Medical University, Saitama, Japan), a graphical user interface for R software (version 2.13.0; The R Foundation for Statistical Computing, Vienna, Austria).^15^

To reduce potential confounding bias between the two groups, propensity score matching (PSM) was performed. Patients in the two groups were matched using the optimal matching algorithm with one-to-one matching without replacement using a caliper of 0.08.

Independent factors that appeared to be significant in the univariate analysis were subsequently assessed using multivariate analysis. Confidence intervals (CIs) were determined at a 95% level. Statistical significance was set at p < 0.05.

## RESULTS

### Comparison of patient characteristics and clinical outcomes before and after PSM

The clinical characteristics of the 131 included patients with esophageal cancer are shown in Table 1. Of these patients, 95 received HMB/Arg/Gln supplementation in the week before esophagectomy (HMB/Arg/Gln group), while 36 did not receive HMB/Arg/Gln supplementation (control group). No patients in the HMB/Arg/Gln group experienced adverse events related to the supplementation. Table 1 shows a comparison of the clinical factors between the two groups before and after PSM, showing that the baseline characteristics were almost similar. Table 2 shows a comparison of the surgical outcomes between the two groups before and after PSM, showing that the outcomes were almost similar.

**Table 1:** Comparison of patient characteristics in the two groups before and after propensity score matching

	Whole cohort			Matched cohort		
	HMB/Arg/Gln group (n = 95)	C group (n = 36)	p value	HMB/Arg/Gln group (n = 36)	C group (n = 36)	p value
Age (yrs), median	68 (38–84)	67 (48–81)	0.861	70 (41–83)	67 (48–81)	0.72
Sex (male/female) (n, %)	79 (83.1)/16 (16.9)	32 (88.8)/4 (11.2)	0.588	31 (86.1)/5 (13.9)	32 (88.8)/4 (11.2)	0.72
cT (1/2/3/4) (n, %)	32 (33.6) 7 (7.4) 49 (51.6) 7 (7.4)	11 (30.6) 2 (5.5) 23 (63.9) 0 (0)	0.971	10 (27.8) 3 (8.3) 22 (61.1) 1 (2.8)	11 (30.6) 2 (5.5) 23 (63.9) 0 (0)	0.97
cN (negative/positive) (n, %)	40 (42.1)/55 (57.9)	18 (50)/18 (50)	0.41	13 (36.1)/23 (63.9)	18 (50)/18 (50)	0.23
cStage (I/II/III/IVa) (n, %)	28 (29.5) 15 (15.8) 45 (47.3) 7 (7.4)	9 (25) 11 (30.6) 16 (44.4) 0 (0)	0.29	10 (27.8) 3 (8.3) 22 (61.1) 1 (2.8)	9 (25) 11 (30.6) 16 (44.4) 0 (0)	0.097
Preoperative treatment CT or CRT/none (n, %)	51 (53.7)/44 (46.3)	24 (66.7)/12 (33.3)	0.17	22 (61.1)/14 (38.9)	24 (66.7)/12 (33.3)	0.62
Height (cm), median	165.9 (149.3–177)	164.3 (149.6–175.9)	0.60	164.9 (151.3–175)	164.3 (149.6–175.9)	0.58
Body weight (kg), median	58.6 (33.7–78.8)	59.9 (42.8–80)	0.22	58.8 (35.7–78.8)	59.9 (42.8–80)	0.12
Body Mass Index (kg/m^2^), median	21.7 (14.4–37.5)	20.8 (14.6–31.2)	0.21	21.6 (14.8–32.7)	20.8 (14.6–31.2)	0.70
Diabetes (n, %)	10 (10.5)	4 (11.1)	0.92	5 (13.9)	4 (11.1)	0.55
Hemoglobin A1c	5.8 (5.4–8.0)	5.8 (5.4–8.0)	0.95	5.8 (5.4–7.5)	5.8 (5.4–8.0)	0.51
Use of steroid agents (n, %)	2 (2.1)	1 (2.8)	0.81	1 (2.8)	1 (2.8)	1.0
Albumin (mg/dL), median	4.0 (3.0–4.7)	3.9 (3.0–5.0)	0.70	4.0 (3.0–4.7)	3.9 (3.0–5.0)	0.38
Total lymphocyte (/μl), median	1666 (602–3173)	1558 (754–3489)	0.36	1617 (602–3045)	1558 (754–3489)	0.15
Total-cholesterol (mg/dL), median	201 (112–297)	198 (150–301)	0.48	207 (104–254)	198 (150–301)	0.83
Onodera PNI	47.8 (34–62.2)	48.8 (33.7–60.3)	0.43	46.1 (35–62.2)	48.8 (33.7–60.3)	0.15
CONUT score	1 (0–6)	1 (0–5)	0.44	1 (0–6)	1 (0–5)	0.18
Hemoglobin, g/dL	10.6 (8.6–15.5)	11.0 (8.3–16.8)	0.82	10.9 (9.0–15.5)	11.0 (8.3–16.8)	0.82

HMB/Arg/Gln group, oral nutrition supplement composed of beta-hydroxy-betamethylbutyrate, arginine, and glutamine group; C group, Control group; cT, clinical tumor invasion; cN: clinical nodal involvement; cStage: clinical stage; CT, chemotherapy; CRT, chemoradiotherapy; PNI, prognostic nutritional index; CONUT, Controlling Nutritional Status.

**Table 2:** Comparison of surgical outcomes in the two groups before and after propensity score matching

	Whole cohort			Matched cohort		
	HMB/Arg/Gln group(n = 95)	C group (n = 36)	p value	HMB/Arg/Gln group(n = 36)	C group (n = 36)	p value
Esophagectomy video-assisted/open (n, %)	89 (93.7)/6 (6.9)	30 (83.3)/6 (16.7)	0.001	30 (83.3)/6 (16.7)	30 (83.3)/6 (16.7)	1.0
Lymph node dissection 2 fields/3 fields (n, %)	20 (21.1)/75 (78.9)	4 (11.2)/32 (88.8)	0.18	6 (16.7)/30 (83.3)	4 (11.2)/32 (88.8)	0.49
Reconstruction gastric tube/ileocolon (n, %)	90 (94.7)/5 (5.3)	32 (88.8)/4 (11.2)	0.23	34 (94.4)/2 (5.6)	32 (88.8)/4 (11.2)	0.23
Anastomosis hand-sewing/circular stapler (n, %)	41 (43.2)/54 (56.8)	16 (44.4)/20 (55.6)	0.89	15 (41.7)/21 (58.3)	16 (44.4)/20 (55.6)	0.81
blood loss (ml), median	194 (0–868)	225 (0–855)	0.052	198 (0–868)	225 (0–855)	0.052
operation time (min), median	429 (295–678)	453 (298–779)	0.071	429 (295–678)	453 (298–779)	0.18
the number of dissected lymph node, median	21 (0–63)	19 (0–40)	0.075	17 (0–45)	19 (0–40)	0.71

HMB/Arg/Gln group, oral nutrition supplement composed of beta-hydroxy-betamethylbutyrate, arginine, and glutamine group; C group, Control group.

Table 3 shows the clinical outcomes for both groups before and after PSM. Of the 131 patients, 36 (27.5%) developed PICs. PICs occurred in 20 patients (21%) in the HMB/Arg/Gln group and in 16 patients (44.4%) in the control group. The rate of PICs was significantly lower in the HMB/Arg/Gln than in the control group (p = 0.007). In this study, no in-hospital deaths occurred.

**Table 3:** Comparison of clinical outcomes between the two groups before and after propensity score matching

	Whole cohort			Matched cohort		
	HMB/Arg/Gln group (n = 95)	C group (n = 36)	p value	HMB/Arg/Gln group (n = 36)	C group (n = 36)	p value
PIC C.D. classification >grade III	20 (21.0%) 10 (10.5%)	16 (44.44%) 10 (27.8%)	0.007 0.014	9 (25.0%) 2 (5.5%)	16 (44.44%) 10 (27.8%)	0.083 0.011
anastomotic leakage	10 (10.5%)	8 (22.2%)	0.082	2 (5.5%)	8 (22.2%)	0.040
pneumonia	18 (18.9%)	8 (22.2%)	0.806	9 (25%)	8 (22.2%)	0.781
recurrent nerve palsy	15 (15.8%)	10 (27.8%)	0.138	7 (19.4%)	10 (27.8%)	0.405
wound infection	3 (3.1%)	1 (2.7%)	0.910	1 (2.7%)	1 (2.7%)	1.0
chylothorax	0 (0%)	1 (2.7%)	0.102	0 (0%)	1 (2.7%)	0.311
postoperative hospital stay	19 (13–155)	21 (13–136)	0.13	18 (13–155)	21 (13–136)	0.596

HMB/Arg/Gln group, oral nutrition supplement composed of beta-hydroxy-betamethylbutyrate, arginine, and glutamine group; C group, Control group; PIC, postoperative infectious complication; C.D. classification, Clavien–Dindo classification.

Regarding patient characteristics, the prevalence of video-assisted esophagectomy was significantly higher in the HMB/Arg/Gln than in the control group (p = 0.001). To reduce potential selection bias between the two groups, PSM analysis was performed using the surgical procedure of esophagectomy and reconstruction as covariates. PSM was used to match 36 patients in the HMB/Arg/Gln group with 36 patients in the control group (Table 1-3). The analysis indicated no differences in patient characteristics, preoperative laboratory data, preoperative nutritional status, or operative factors between the two groups. The rates of anastomotic leakage (5.5% vs. 22.2%; p = 0.04) and CD classification grade III or higher PIC (5.5% vs. 27.8%; p = 0.011) were significantly lower in the HMB/Arg/Gln than in the control group.

### Evaluation of wound healing time in anastomotic leakage cases

Anastomotic leakage occurred in 10 patients in the HMB/Arg/Gln group and in 8 patients in the control group. We investigated the healing time of anastomotic leakage. Healing time was defined as the number of days from the diagnosis of anastomotic leakage to the closure of the fistula. The healing time was shorter in the HMB/Arg/Gln group (18 days, range 7–25 days) than in the control group (25 days, range 21–56 days) (p = 0.033).

## DISCUSSION

This study demonstrated that preoperative HMB/Arg/Gln supplementation can help reduce anastomotic leakage and the aggravation of PIC. Second, it may also improve the healing of anastomotic leakage. To the best of our knowledge, this study is the first to demonstrate the role of HMB/Arg/Gln in esophageal surgery.

Despite recent advancements in surgical procedures and perioperative management, esophagectomy remains associated with high morbidity and mortality rates.^7^^,^^16^ Postoperative complications have been shown to affect long-term outcomes after esophagectomy.^7^ Esophageal cancer is often characterized by progressive dysphagia, which places patients at a substantial risk of malnutrition.^17^^,^^18^ Therefore, preoperative interventions are important for preventing postoperative complications. Among these, nutritional management is one of the most important factors in preventing severe postoperative complications and enhancing supportive care.

An oral nutrition supplement composed of HMB, Arg, and Gln has been reported to improve wound healing by stimulating protein and collagen synthesis [8, 19–22].^8^^,^^19–22^ Yokota et al. reported the potential to reduce the incidence of chemoradiotherapy-induced severe oral mucositis and promote rapid recovery.^9^ Gln and Arg have been shown to enhance collagen synthesis in vitro and improve T cell-mediated immune function.^23–25^ HMB stimulates protein synthesis and attenuates proteolysis.^26^ Therefore, our results suggest that HMB/Arg/Gln supplementation, a mixture of these three nutrients, may accelerate wound healing and prevent the aggravation of complications.

Additionally, HMB/Arg/Gln supplementation may reduce anastomotic leakage and shorten its duration. Anastomotic leakage, a major complication following esophagectomy, has been reported in 10%–25% of cervical anastomoses.^27^ However, the most effective treatment for anastomotic leakage remains controversial,^27^^,^^28^ with no defined regimen for its management.^29^^,^^30^ Treatment options range from close observation to surgical re-intervention. Surgical approaches for repair and drainage via tube placement are selected for less severe leaks, while complete gastrointestinal diversion is used for extensive disruption.^31^^,^^32^ Endoscopic stenting has also been reported as successful.^32^ However, the most important factor is the prevention of anastomotic leakage. HMB/Arg/Gln supplementation could play a role in preventing severe postoperative complications.

Regarding adverse events associated with HMB/Arg/Gln supplementation, increases in blood urea nitrogen (BUN) and instances of diarrhea have been reported.^9^^,^^33^^,^^34^ In our study, patients receiving HMB/Arg/Gln showed elevated BUN levels independent of blood creatinine levels (data not shown), and no severe cases of diarrhea were observed. Both the increase in BUN levels and the diarrhea were manageable. However, the timing of the supplementation warrants discussion. HMB/Arg/Gln was administered only during the preoperative period due to safety concerns surrounding Arg in the perioperative period. Arg administration has been associated with increased mortality in patients with severe sepsis and those in intensive care units.^35^^,^^36^ Esophagectomy is a highly invasive procedure, often leading to serious postoperative complications such as pneumonia and anastomotic leakage, which can result in multiorgan failure. Arg induces the overproduction of nitric oxide during severe inflammation, and nitric oxide-related cytotoxicity poses a risk for post-esophagectomy patients. Thus, to mitigate these risks, HMB/Arg/Gln supplementation was limited to the preoperative period and was avoided postoperatively.

The present study was conducted by the single-center retrospective design and the relatively small study sample. This study had limitations that should be considered when interpreting the findings. First, it employed a single-center retrospective design. The observed results may be partially influenced by random error due to the small sample size. Second, the optimal dose of supplementation for each case was not defined. Therefore, future research should investigate the effects of HMB/Arg/Gln supplementation. We consider the replication in independent cohorts is important as a critical next step to confirm the robustness of our findings.

In conclusion, we found that HMB/Arg/Gln supplementation was associated with reduced risk of anastomotic leakage and PIC severity following esophagectomy. Further studies are required to determine the effects of HMB/Arg/Gln.

## CREDIT TAXONOMY

Katsushi Takebayashi (Conceptualization, Data curation, Formal analysis, Writing—original draft, Writing—review & editing), Sachiko Kaida (Writing—review & editing), Reiko Otake (Writing—review & editing), Asuka Fukuo (Writing—review & editing), Toru Miyake (Writing—review & editing), Masatsugu Kojima (Writing—review & editing), Soichiro Tani (Writing—review & editing), Hiromitsu Maehira (Writing—review & editing), Haruki Mori (Writing—review & editing), Hajime Ishikawa (Writing—review & editing), Masaji Tani (Writing—review & editing).
